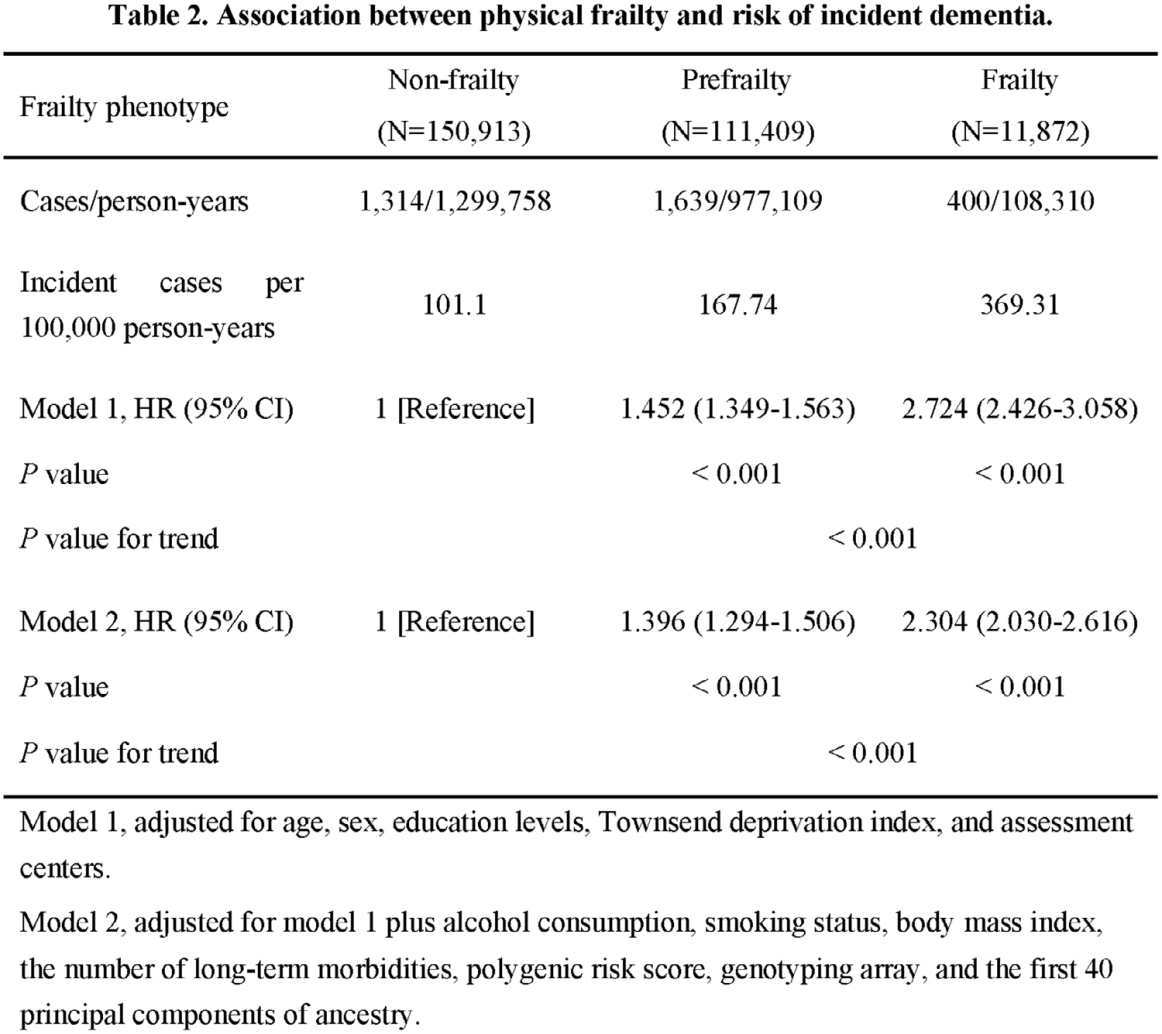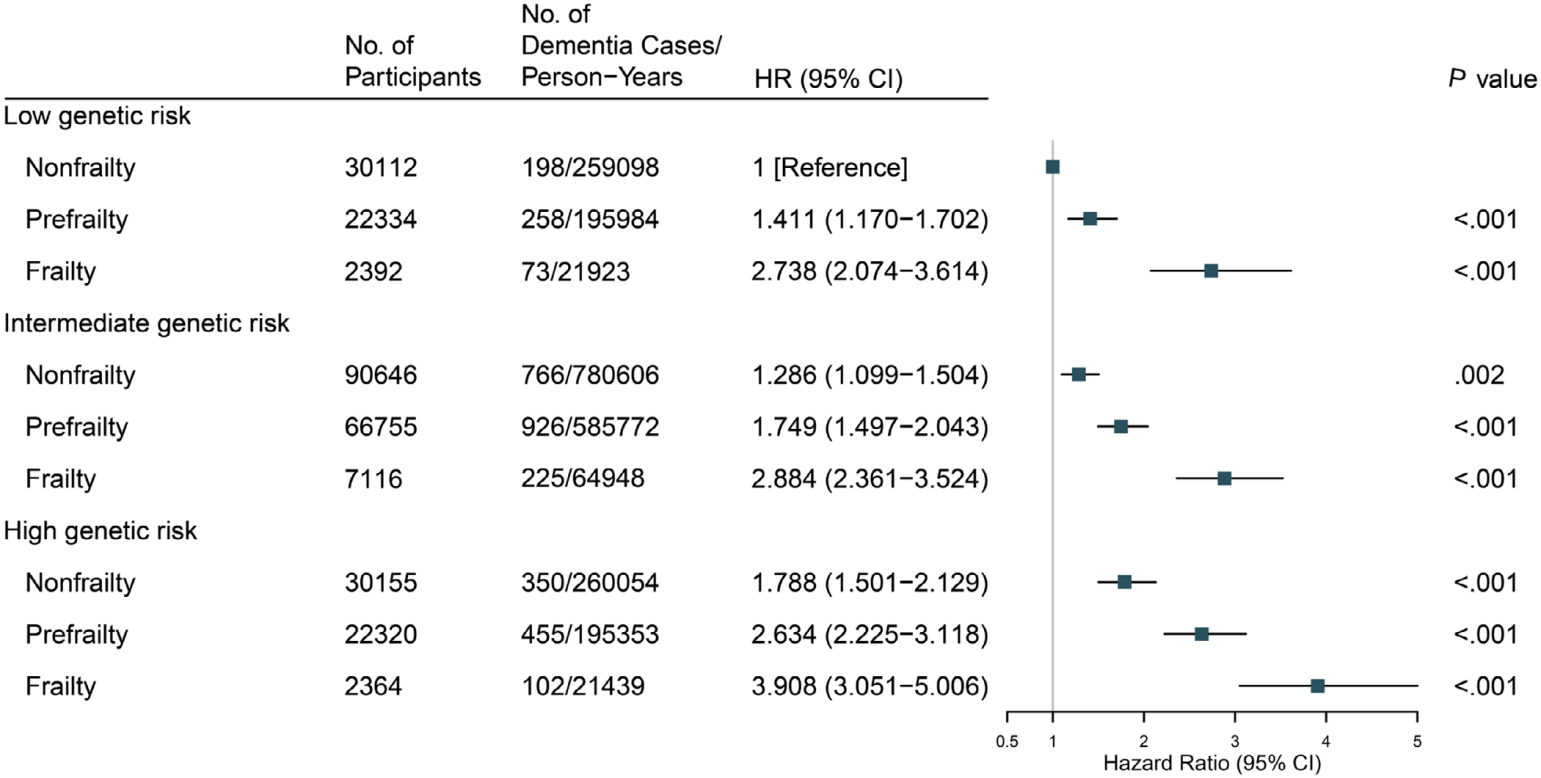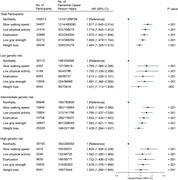# Physical frailty, genetic predisposition, and incident dementia: a prospective cohort study of 274,194 participants

**DOI:** 10.1002/alz.085929

**Published:** 2025-01-09

**Authors:** Pei‐Yang Gao, Lan Tan, Jin‐Tai Yu

**Affiliations:** ^1^ Qingdao Municipal Hospital, Qingdao University, Qingdao China; ^2^ Qingdao Municipal hospital, Qingdao university, Qingdao, Shandong China; ^3^ Huashan Hospital, Fudan University, Shanghai, Shanghai China; ^4^ National Center for Neurological Disorders, Shanghai China

## Abstract

**Background:**

Frailty, a condition characterized by functional decline, is recognized as a potential modifiable risk factor for dementia prevention. Genetic risk is also considered an unregulated risk for dementia. However, their joint effect remains unclear. Therefore, this study aims to investigate the long‐term associations of physical frailty, genetic predisposition, and their combined effect on dementia risk.

**Method:**

Participants without baseline dementia from the UK Biobank were included in this prospective longitudinal cohort study. Physical frailty was measured by the frailty phenotype, which comprises five components: slow walking speed, low physical activity, exhaustion, low grip strength, and weight loss; and a polygenic risk score (PRS) for dementia with low, intermediate, and high risk categories. Cox proportional hazards regression models were used to determine the association between physical frailty and genetic and dementia risks.

**Result:**

Of the 274,194 participants (females, 146,574 [53.45%]; mean age, 57.24), 3,353 new‐onset dementia events were documented. Compared to non‐frailty, the hazard ratio (HR) for dementia incidence in pre‐frailty and frailty was 1.396 (95% confidence interval [CI], 1.294–1.506, *P* < 0.001) and 2.304 (95% CI, 2.030‐2.616, *P* < 0.001), respectively. Compared to non‐frailty and low PRS, the HR for dementia risk was 3.908 (95% CI, 3.051‐5.006, *P* < 0.001) for frailty and high PRS, which was monotonically increasing risk of incident dementia across the frailty status and PRS categories. Furthermore, slow walking speed (HR, 1.817; 95% CI, 1.640‐2.014, *P* < 0.001), low physical activity (HR, 1.719; 95% CI, 1.545–1.912, *P* < 0.001), exhaustion (HR, 1.670; 95% CI, 1.502–1.856, *P* < 0.001), low grip strength (HR, 1.606; 95% CI, 1.479–1.744, *P* < 0.001), and weight loss (HR, 1.464; 95% CI, 1.328–1.615, *P* < 0.001) were independently associated with dementia risk compared to non‐frailty. Additionally, precise differences among different dementia genetic risk populations were also identified due to differences in dementia risk resulting from the constitutive patterns of frailty.

**Conclusion:**

Both physical frailty and high genetic risk are significantly associated with an increased risk of dementia. Early intervention to modify frailty is beneficial for achieving primary and precise prevention of dementia, particularly in those with a high genetic risk.